# Emerging Role of Alarmins in Food Allergy: An Update on Pathophysiological Insights, Potential Use as Disease Biomarkers, and Therapeutic Implications

**DOI:** 10.3390/jcm12072699

**Published:** 2023-04-04

**Authors:** Angela Rizzi, Elena Lo Presti, Raffaella Chini, Luca Gammeri, Riccardo Inchingolo, Franziska Michaela Lohmeyer, Eleonora Nucera, Sebastiano Gangemi

**Affiliations:** 1 UOSD Allergologia e Immunologia Clinica, Dipartimento Scienze Mediche e Chirurgiche, Fondazione Policlinico Universitario A. Gemelli IRCCS, 00168 Rome, Italy; angela.rizzi@policlinicogemelli.it (A.R.); raffaella.chini01@gmail.com (R.C.); eleonora.nucera@policlinicogemelli.it (E.N.); 2 Institute for Biomedical Research and Innovation, National Research Council of Italy (IRIB-CNR), 90146 Palermo, Italy; elena.lopresti@irib.cnr.it; 3 Department of Clinical and Experimental Medicine, School and Operative Unit of Allergy and Clinical Immunology, University of Messina, 98125 Messina, Italy; lucagammeri@outlook.com (L.G.); gangemis@unime.it (S.G.); 4 Pulmonary Medicine Unit, Department of Neurosciences, Sense Organs and Thorax, Fondazione Policlinico Universitario A. Gemelli IRCCS, 00168 Rome, Italy; 5 Direzione Scientifica, Fondazione Policlinico Universitario A. Gemelli IRCCS, 00168 Rome, Italy; franziska1.lohmeyer@gmail.com; 6 Medicina e Chirurgia Traslazionale, Università Cattolica del Sacro Cuore, 00168 Rome, Italy

**Keywords:** food allergy, damage-associated molecular patterns, nuclear alarmins, granule derived alarmins, cytoplasmic alarmins, IgE mediated food allergy, non-IgE mediated food allergy, eosinophilic esophagitis

## Abstract

Food allergies are immuno-mediated adverse reactions to ingestion or contact with foods, representing a widespread health problem. The immune response can be IgE-mediated, non-IgE-mediated, or with a mixed mechanism. The role of innate immunity and alarmins in the pathogenesis of diseases such as asthma and atopic dermatitis is well known. Some authors have investigated the correlation between alarmins and food allergies, often obtaining interesting results. We analyzed articles published in English from the last 22 years present on PubMed concerning the role of alarmins in the pathogenesis of food allergies and their potential use as disease biomarkers, response biomarkers to therapy, or potential therapeutic targets. Nuclear alarmins (TSLP, IL-33, IL-25) appear to have a critical role in IgE-mediated allergies but are also implicated in entities such as eosinophilic esophagitis. Calprotectin and defensins may play a role as disease biomarkers and could help predict response to therapy, although results in the literature are often conflicting. Despite the promising results, more studies on humans still need to be conducted. Deepening our knowledge regarding alarmins and their involvement in food allergies could lead to the development of new biological therapies, significantly impacting patients’ quality of life.

## 1. Introduction

Food allergy (FA) is defined as an immune-mediated adverse reaction after ingesting or coming into contact with harmless food allergens [1,2]. The prevalence of food allergies represents a major global health problem, more commonly affecting children rather than adults [3,4,5] with dramatically increasing rates of food allergy-associated hospitalizations [6,7].

Uncertain epidemiological details are caused by difficulties documenting food allergy because food intolerances (less-serious adverse reactions but sometimes with similar symptoms) are often mistaken for food allergies [3]. Census to identify food allergies is complicated by the diverse diagnostic processes used in different countries, which rely on medical history, tests for sensitization, and oral food challenges of patients of all ages [8]. However, epidemiological studies in several parts of the world confirm the relevant prevalence of FA in infants and school-aged children [9], and around 40% of children with FA are allergic to more than one food [1]. The incidence of FA has steadily increased over time, suggesting that environmental influences may promote food allergy. Indeed, the current guidelines from the European Academy of Allergy and Clinical Immunology (EAACI) recommends approaches to prevent the development of FA for children from 1 year up to 5 years [10]. A common suggestion is to avoid some food allergens, such as cow’s milk as a supplement for breastfed infants in the first weeks of life or to introduce only well-cooked eggs as part of complementary feeding. Moreover, the EAACI suggests avoiding dietary food allergens during pregnancy or breast-feeding, using soy protein in the first 6 months of life. For adults, the main common allergens are eggs, milk, peanuts, tree nuts, soy, wheat, crustacean shellfish, and frequently consumed fish [11]. Experimental models focusing on genetic, epigenetic, and environmental risk factors reveal that a complex interplay between the epithelial barrier, systemic and mucosal immune responses, exposure pathways, and the microbiome may favor allergy or tolerance [1]. The central construct driving FA modeling is based on perturbations of homeostasis at barrier sites induced by food antigens. Perturbations can activate or lower the threshold activation of the immune system. This process is the major cause of allergic sensitization. However, a deep understanding of these early steps that trigger sensitization remains unclear because sensitization is a silent process [12].

The main purpose of maintaining homeostasis is food quality control, and these protective mechanisms are mediated by CD4+ Th2 cells and IgE, which are the principal effector molecules in food-induced type 2 responses, although the uncontrolled type 2 immune response also encompasses non-IgE-mediated diseases.

Allergen-specific IgE is bound to tissue-resident mast cells and circulating basophils through its high-affinity receptor, which is called FcεRI. Upon re-exposure, the allergen crosslinks surface-bound IgE and triggers mast cell and basophil degranulation, which causes acute allergic reactions, including anaphylaxis.

**Figure 1:**
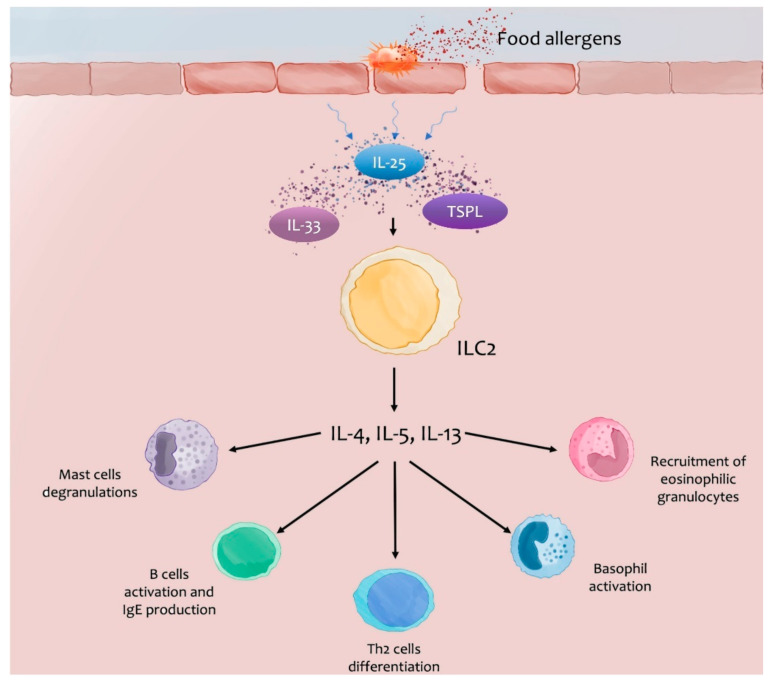
The damaged skin barrier or intestinal mucosa allows the passage of food allergens and subsequent sensitization. Furthermore, the damage causes the release of alarmins (IL-25, IL-33, and TSLP) that activate the ILC2 with the expansion and release of Th2 proinflammatory cytokines (IL-4, IL-5, and IL-13) [13].

Innate immune cells with activity Th2-like (ILC2) participate in the regulation of homeostasis in a complex interaction with secreting molecules named alarmins (Figure 1) [13]. Epithelial and endothelial cells are the first cells reacting to tissue perturbations by producing molecules such as IL-33, IL-25, and thymic stromal lymphopoietin (TSLP). These alarmins bind receptors on ILC2, which expand, and at the same time secrete IL-5 (a facilitator of eosinophils accumulation at the tissue site), IL-13 (a factor permitting the switch in the IgE immunoglobulin production), and amphiregulin with a pleiotropic role. IL-25 and IL-33, derived from intestinal epithelial cells, activate Th2 cells, which release IL-4, with effects on the Th2 cells itself. This process has the role of amplifying mastocytes and basophil activation that, as a positive feedback loop, act on the Th2-like immune response. More precisely, alarmins induce the expression of CCR7 on basophils that can migrate in the paracortical section of lymph nodes, activating IL-4-producing basophils. Experimental studies suggest that cutaneous basophil infiltration could guide the recruitment of eosinophils and promote food antigen sensitization, with the consequent development of IgE-mediated FA [14].

Recently, two newly identified Th2A and Tfh13 populations seem to be closely related to allergen-specific T cell phenotypes, contributing to IgE memory responses [15]. Briefly, Th2A expresses the Th2-related markers CRTH2, CD200R, and CCR4; secrete Th2 cytokines; and have very poor proliferation capacity [16,17]. This population is present only in allergic individuals with up-expression of specific markers [16]. However, their role is still unclear because of doubts regarding the function of Th2 in IgE production by B cells. Indeed, Tfh13 cells appear to be involved in IgE production by B cells [18]. They are IL-13-secreting Tfh (T follicular helper) cells (Tfh13) that regulate high-affinity IgE production, as demonstrated in mice and allergic patients [19]. Definitively, many other studies need to demonstrate their role in human allergies and specifically in food allergies.

Innate cells contribute to food allergies [20]. Children with persistent egg allergy present a unique innate immune signature, where there are increasing numbers of circulating inflammatory cytokines-producing myeloid cells [21].

Alarmins are a relatively new term in immunology that emerged from a proposal by Dr Joost Oppenheim at the EMBO Workshop on Innate Danger Signals and High-mobility group box (HMGB) in February 2006 [22,23]. They are also called “damage-associated molecular patterns, DAMPs”, first described 2 years earlier by Seong and Matzinger [24]. HMGB1 is involved in inflammation and oxidative stress; targeting HMGB1 could lead to new therapeutic strategies [25].

Alarmins can be grouped into three main categories: (1) nuclear, including high mobility group protein B1 (HMGB-1), high mobility group protein N1 (HMGN1), IL-33, and IL-1α; (2) granule derived, including α- and β-defensins, cathelicidin (LL37/cathelicidin-related antimicrobial peptide (CRAMP), eosinophil-derived neurotoxin (EDN), and granulysin; and (3) cytoplasmic, such as heat-shock protein (HSP-60, -70, -90, and -96), S100 proteins, ATP, and uric acid [26].

Recently, many reports emphasizing the important role of alarmins have been published. These cytokines are of epithelial origin and are triggered by, among others, damaging factors, viral infections [27], and allergic inflammation. These observations support the hypothesis that food allergy develops through transcutaneous sensitization in children with an impaired skin barrier [28].

Alarmins signal the innate immune system to initiate reparative functions to regain homeostasis. Furthermore, a link between innate immune–alarmins–memory allergy exists. In the last decade, intense studies on memory cells maintaining food allergies for life have been conducted [15]. Indeed, in the draining lymph nodes, Th0 interact with migratory dendritic cells (DCs), which present food allergens captured on site (mucosa or skin) [29]. This is possible because alarmins and other innate-stimulatory factors have activated DC during allergen capture [30,31]. The expression of co-stimulator molecules such as OX40L on APC and its ligation with OX40 on T cells induce Th2 polarization. This is only the starting point of the exchanging signals. The avidity and the duration of TCR-MHC interaction will influence memory Th2 cell survival, promoting IL2 responsiveness and IL4R expression on Th2 [32]. The robust clonal expansion of Th2 cells empowers survival and permits the generation of memory T cells against food allergies, with the participation of Th2A and Thr13 [33,34].

It has been speculated that IL-9 is the propulsive engine to stepwise develop and progress FAs. In an autocrine loop, mucosal mast cells produce IL-9, but ILC2s and Th9 cells may also serve as alternative cellular sources of this cytokines to amplify the response. Some studies have demonstrated that alarmins (TSLP and IL-25) can promote Th9 cell generation and function [35]. Th2- and Th9-associated genes were increased in children with peanut allergy. Moreover, it has been speculated that the IL9 expression level could be an indicator to distinguish patients with a peanut allergy from those with peanut sensitization [36].

In this report, we review the role of alarmins in the pathophysiology of food allergies, including IgE-mediated food allergy (such as cow’s milk allergy (CMA)), non-IgE mediated food allergy (such as non-IgE cow’s milk protein allergy (CMPA), food protein-induced allergic proctocolitis (FPIAP), and food protein-induced enterocolitis syndrome (FPIES)) and mixed IgE cellular-mediated food allergy (such as eosinophilic esophagitis).

## 2. Search Strategy

This research was conducted using the international database PubMed; the search strategy was limited to articles published between 1 January 2000 and 28 February 2022. Original articles were included in the evaluation, whereas review articles, conference papers, and articles outside the scope of the review were excluded. Only articles published in English were considered.

The keywords used were: “alarmins” OR “nuclear alarmins” OR “TSLP” OR “IL-33” OR “IL-25” OR “IL-1α” OR “HMGB1” OR “HMGN1” OR “granule-derived alarmins” OR “defensins” OR “defensins α” OR “defensins β” OR “eosinophil-derived neurotoxin” OR “granulysin” OR “cytoplasmic alarmins” OR “S100 proteins” OR “calprotectin” OR “Heat-Shock proteins” OR “HSP” OR “ATP” OR “adenosine 5′-triphosphate” OR uric acid” AND “food allergy” OR “IgE mediated food allergy” OR “milk allergy” OR “egg allergy” OR “peanut allergy” OR “shrimp allergy” OR “alpha-gal allergy” OR “non-IgE mediated food allergy” OR “non-IgE CMPA” OR “FPIAP” OR “FPIES” OR “food-protein enteropathy (FPE)” OR “Food-induced pulmonary hemosiderosis (Heiner syndrome)” OR “cow’s milk-induced anemia” OR “mixed food allergy” OR “eosinophilic esophagitis” OR “dermatitis herpetiformis”. Finally, we selected articles in which the role of one or more alarmins in previously mentioned food allergies had been investigated, and we grouped papers into studies on humans and animal models.

## 3. Results

Human studies investigating nuclear, granule-derived, and cytoplasmic alarmins in food allergy, published in the last two decades, are displayed in Table 1, Table 2, and Table 3, respectively.

### 3.1. IgE-Mediated FA

IgE-mediated sensitization to foods often occurs in infancy without a known prior oral exposure, suggesting that alternative exposure routes contribute to food allergy. It is also well known that atopic dermatitis (AD) and food allergy are closely linked in the so-called “atopic march”, demonstrating that a disrupted skin barrier is a potential factor in the increasing prevalence of food allergy. These data suggest that cutaneous sensitization with a food antigen before its consumption elicits the development of food allergy via the innate immune system through cytokines, the so-called alarmins (TSLP, IL-25, IL-33), which are directly released from damaged epithelium and contribute to sensitization to foods in early life.

In the literature, several studies in murine models and in humans were conducted with the purpose of demonstrating the role of these innate cytokines in the pathogenesis of IgE-mediated FA from the phase of sensitization to food antigens to the risk of anaphylaxis on rechallenge with the same food.

#### 3.1.1. Nuclear Alarmins: Thymic Stromal Lymphopoietin (TSLP), IL-33, IL-25

##### Studies on Animal Models

The functional role of TLSP in food allergy emerged in studies on mouse models [37]. In 2010, Blázquez et al. [37] studied TSLP receptor TSLPR+/+ and TSLPR−/− mice that were sensitized and challenged with ovalbumin (OVA) using models of allergic diarrhea or systemic anaphylaxis. They observed that TSLPR−/− mice were protected from the onset of allergic diarrhea, showed reduced ovalbumin-IgEs in their serum, and lost Th2-cells in the jejunum compared with TSLPR+/+ mice.

Subsequently, Noti et al. [38] revealed that mice, epicutaneously sensitized with OVA or peanut on AD-like skin lesion followed by intragastric antigen-specific challenge, showed expanded TSLP-elicited basophils in the skin, promoting antigen-specific Th2 cytokine responses, elevated antigen-specific serum IgE levels, and accumulated mast cells in the intestine, favoring the development of intestinal food allergy. Moreover, disruption of TSLP responses or basophil depletion reduced the susceptibility to intestinal FA, while transfer of TSLP-elicited basophils into intact skin promoted the development of allergic disease [38].

Subsequent studies revealed that TSLP is an essential, but not exclusive, mediator for elicitation of FA in mice [39].

IL-33 drives Th2-associated cytokine production in children [40] and adults [41] with allergic asthma. In addition, some authors have demonstrated its role in food allergy.

In 2016, Galand et al. [42] demonstrated that IL-33 is released following mechanical skin injury, enhances IgE-mediated mast cell degranulation, and promotes oral anaphylaxis following epicutaneous sensitization in AD murine models.

Subsequently, Khodoun et al. [43] showed that IL-33-driven allergic disease occurred in TSLP-independent disease, and mice lacking IL-33 signaling were protected from the onset of allergic diarrhea in TSLP-driven disease. Furthermore, the specific loss of IL-33 expression in the epithelium attenuated cutaneous inflammation [43].

These data reveal that IL-33 plays a critical role during early cutaneous inflammation and during challenge. The exposure of mice to different food allergenic extracts (peanuts, OVA, etc.) on undamaged areas of skin or on AD skin lesions led to sensitization and anaphylaxis upon rechallenge with the same food antigens. In particular, epicutaneous peanut protein extract exposure on undamaged areas of skin induced sensitization to the peanut components Ara h 1 and Ara h 2, as well as in human peanut allergy [44]. It seems that topical peanut extract application induced an alteration dependent on the IL-33 receptor ST2 in skin-draining DCs, resulting in Th2 cytokine production from T cells [44].

Recently, the role of the ST2 IL33 receptor on CD4+ T cells was emphasized by Pérez-Rodríguez et al. [45], who evaluated the role of egg yolk (EY) in the induction of allergy to egg white (EW) in murine models. The intragastrical exposure to a mixture of EW:EY induced higher expansion of CD4+ T cells expressing ST2—IL-33 receptors than that observed with either EW or EY added individually [45].

Among alarmins, it seems that TSLP and IL-33 have slightly different roles in mouse models of FA. TSLP has a pivotal role in Th2 responses during the sensitization phase of FA., while IL-33 is essential for inducing IgE-dependent anaphylaxis in the gut [46].

Similar to TSLP, IL-25 seems to have a role in the initiation of type 2 immunity in the intestine [47]. In murine models of AD, mice, which have been epicutaneously sensitized with antigens and lacked the IL-25 receptor, failed to develop acute diarrhea and anaphylaxis [47].

##### Studies on Humans

The role of TSLP, IL-33, and IL-25 in the pathophysiology of IgE-FA is also supported by studies on humans.

In 2021, Ukleja-Sokołowska et al. [48] described the first analysis of TSLP, IL-33, and IL-25 in a group of adult patients with FA symptoms related to shrimp consumption. In a cohort of thirty-seven patients, the concentrations of blood serum TSLP and IL-25 were significantly higher than in the control group.

In the same year, Paparo et al. [49] explored the tolerogenic effect elicited by the protein fraction of different formulas available for the dietary treatment of CMA and found that the production of TSLP and IL-33 was significantly increased by extensively hydrolyzed whey formula (EHWF), hydrolyzed rice formula (HRF), and soy formula (SF). These data suggest a role of TSLP and IL-33 in the induction of food tolerance in humans.

Interestingly, the production of Th2-stimulating epithelial factors, including TSLP and IL-33, is linked to some oral microbial species. In a human multi-omic study of the oral environment in FA, Ho et al. [50] found that oral *Prevotella* spp. abundances were correlated with decreased local secretion of TSLP and IL-33, while oral *Neisseria* spp. abundance was positively associated with an increased Th2-cytokines production.

Finally, Aalberse et al. [51] investigated the behavior of IL-25, the most divergent member in the IL-17 family, in a well-defined cohort of peanut sensitized children undergoing a double-blind placebo-controlled food challenge (DBPCFC). Plasma IL-25 was elevated only in the subgroup of children with a positive DBPCFC outcome. On the contrary, plasma IL-25 was absent in children with a negative DBPCFC outcome and in healthy controls. This suggests that elevated plasma IL-25 may be a sign of a severe atopic phenotype [51].

#### 3.1.2. Granule Derived Alarmins: Eosinophil-Derived Neurotoxin (EDN)

##### Studies on Humans

Eosinophils are activated in FA intestinal inflammation [52], and previous studies have shown that EDN, one of the major proteins released by eosinophils, might serve as a possible marker of ongoing food allergic reactions [52,53].

In 2012, Kalach et al. [54] explored the diagnostic performances of fecal markers, including EDN, in comparison with the standard allergic work-up in a heterogeneous group of children referred for CMA diagnosis, with only three patients with positive IgE-cow’s milk protein titers.

In 2016, Winberg et al. [55] explored the potential prognostic role of fecal biomarkers, analyzing cytokine mRNA expression for the 13 cytokines involved in humoral Th2-, cytotoxic Th1-, regulatory Th3/Tr1-, and inflammatory responses. The authors found that the median level of fecal EDN at baseline was almost twice as high in children with a positive challenge compared to a negative challenge outcome, even though the results were not statistically significant [55].

Two years later, Salmivesi et al. [56] explored the possible prognostic role of 15 allergy, immunology, or inflammatory parameters, including EDN, in 28 adolescents with cows’ milk allergy after six-months of oral immunotherapy (OIT) protocol.

#### 3.1.3. Cytoplasmic Alarmins: S100, Calprotectin, Adenosine 5′-Triphosphate (ATP), Heat-Shock Protein (HSP), Uric Acid

##### Studies on Animal Models

Calprotectin, also known as S100A8/A9, has a confirmed role in intestinal inflammation. To investigate the role of calprotectin and inflammatory-related factors (TLR4, NF-κB, TNF-α, IL-6, and IL-1β) in food allergy, Zhu et al. [57] selected 80 3-week-old male Brown Norway rats: 40 rats were randomly assigned to the OVA-sensitized experimental group, while 40 rats were assigned to the normal saline sham-sensitized control group. Compared with the control group, the experimental group had statistically significant higher serum levels of S100A8/A9, TLR4, TNF-α, NF-κB, IL-1β, and IL-6 at specific study timepoints (all *p* < 0.05). Moreover, positive correlations were found between the serum levels of S100A8/A9 and inflammation-associated cytokines (TNF-α: r = 0.378, *p* = 0.039; IL-1β: r = 0.679, *p* = 0.000; IL-6: r = 0.590, *p* = 0.001).

The study confirmed the preliminary results of a previous study by the same authors in a similar model of FA [58] in which the influence of calprotectin on CD4+ T and dendritic cells was also observed by co-culturing CD4+ T cells with dendritic cells, which revealed a shift toward increased Th2 T cells in calprotectin-treated cultures.

These studies demonstrate that calprotectin and inflammatory-related factors (TLR4, NF-κB, TNF-α, IL-6, and IL-1β) promote the inflammation seen in FA, which is considered the result of a complex interaction between immune and inflammatory factors [32].

Adenosine 5′-triphosphate (ATP) is released from the cytoplasm under physiologic and pathophysiologic conditions and enters the extracellular space, where it acts on a group of cell-surface receptors termed P2-purinoceptors [59]. In 1970, Burnstock et al. [60] first demonstrated that ATP is involved in manipulating gastrointestinal motility as a non-adrenergic, non-cholinergic neurotransmitter. Nowadays, ATP is known to regulate intestinal motility as an excitatory and inhibitory neurotransmitter in different species and different intestinal segments through P2-purinoceptors [61]. Furthermore, it is released from peritoneal mast cells, while mucosal mast cells release it by antigen stimulation [62]. Finally, ATP ameliorates anti-IgE-induced human lung mast cell degranulation [63].

In 2008, Leng et al. [64] explored the possible pathophysiological role of ATP in a murine model of FA with disturbed intestinal motility. The authors found that the sustained alteration in cholinergic, purinergic, and sensory neuronal circuitry during the chronic intestinal anaphylaxis contributes to the development of motility malfunction in FA [64].

##### Studies on Humans

Also in humans, calprotectin seems to be closely linked to IgE-mediated food hypersensitivity. In a prospective case–control trial enrolling 90 milk-allergic infants, fecal calprotectin level in the allergic group (median: 410 μg/g) was significantly higher than in the non-allergic group (median: 141 μg/g) (*p* < 0.001) [65]. Moreover, after dietary interventions, fecal calprotectin levels of the infants with a milk protein allergy were significantly lower than those before the intervention (*p* < 0.001), while the growth index values (weight and length) were significantly higher than those before dietary intervention (*p* < 0.05) [65].

In a previous study, Carroccio et al. [66] investigated the role of calprotectin to support the diagnose of FA, and observed that twenty-five percent of patients with irritable bowel syndrome-like symptoms are affected by food hypersensitivity. These patients had increased levels of fecal calprotectin, as well as ECP and tryptase, indicating that they might cause inflammation in patients with irritable bowel syndrome [66].

Subsequently, in the previously mentioned study, Winberg et al. [55] demonstrated increased fecal calprotectin levels in challenge-proven FA compared to a negative challenge, although they were non-statistically significant.

From these studies it emerged that fecal calprotectin could be used as a biomarker to diagnose and monitor food allergies in patients because its levels correlate with the inflammation and the immune responses to food antigens.

Heat-Shock proteins (HSP) are a family of proteins produced by cells in response to exposure to stressful conditions. Hsp70 is one of the representative members of the HSPs family, and some studies have reported that elevated Hsp70 expression inhibits the production of pro-inflammatory cytokines in various cell types via preventing NF-κB activation [67,68,69].

In 2002, Derebery [70] investigated the prevalence of elevated HSP-70 in patients with Meniere’s disease who had a milk allergy compared with those who were not allergic to milk. In this comparative study, the prevalence of HSP-70 elevation was actually lower in patients with milk allergy than in those without milk allergy, although it was not statistically significant. Furthermore, the prevalence of HSP-70 elevation was substantially lower in younger (<50 years) relative to older (≥50 years) patients [70].

Subsequently, Nair et al. [71] compared the proteomic profiles of radioallergosorbent test (RAST+) patients with severe food allergies and RAST− patients using 2D-DIGE analysis to obtain candidate biomarkers specific to food allergies. The researchers highlighted a group of key proteins that play a role in FA. These included heat shock proteins that were significantly lower in RAST+ patients compared to RAST− patients (HSP-40 homolog: 19% decrease) [71].

Uric acid is one of the damage-associated molecular pattern molecules (DAMPs) released from stressed or damaged cells [26,72,73]. These molecules act as endogenous danger signals that alert the innate immune system to unscheduled cell death and microbial invasion [72,73,74], activate eosinophil functions such as degranulation and cytokine production [75], and play important roles in allergic disorders [76].

In 2016, Min and Min [77] estimated the potential risk of hyperuricemia in a population of 3893 adults in the USA according to the presence of milk allergen sensitization based on IgE-mediated sensitivity to milk. Data were obtained from the 2005–2006 National Health and Nutrition Examination Survey (NHANES), conducted by the Centers for Disease Control and Prevention. The authors found that adults with sensitization to milk allergen were at higher risk for increased uric acid (β = 0.29; 95% CI = 0.09–0.48) and hyperuricemia (OR = 2.08; 95% CI = 1.18–3.68) compared with adults not sensitized to milk allergen, supporting the hypothesis that sensitization to cows’ milk allergen might be a risk factor for increased serum uric acid [77].

### 3.2. Non-IgE-Mediated FA

#### 3.2.1. Non-IgE Cow’s Milk Protein Allergy (NICMPA)

Cow’s milk protein (CMP) is the main cause of FA in the first year of life. Based on the involvement of IgE antibodies, CMP allergy (CMPA) is classified as a classic IgE-mediated, non-IgE cow’s milk protein allergy (NICMPA), with a mixed pathophysiology [78].

##### Granule Derived Alarmins: Defensins (α, β), EDN

Studies on humans

In a prospective study including 57 infants and young children (median age 8.7 months) with gastrointestinal symptoms in NICMPA, Merras-Salmio et al. [79] analyzed serum and stool samples collected during a cow milk protein-free diet and after both placebo and active-food challenges. Compared to the control group, fecal β-defensin 2, as well as IgA levels, showed high levels of within-group variation and higher median levels in DBPCFC-positive infants, even if not statistically significant [79].

In 2007, Wada et al. [80] analyzed in detail the sequential changes of stool parameters in a female newborn with NICMPA and found fecal EDN increase during the oral challenge test. This result suggest that eosinophils played some role during the acute phase and in the subsequent development of the chronic inflammation in this case report [80].

##### Cytoplasmic Alarmins: S100, Calprotectin

Studies on humans

Different studies have investigated the possible diagnostic and prognostic roles of fecal calprotectin in patients with NICMPA.

In 2014, Beşer et al. [81] found statistically significant differences in fecal calprotectin values between IgE-mediated and non-IgE-mediated groups, either before or after a CMP elimination diet (*p* = 0.001 and *p* = 0.025, respectively).

In the same year, Merras-Salmio et al. [79] found higher fecal calprotectin values in patients following an elimination diet with a positive challenge than in patients with a negative challenge performed with CMP, demonstrating the presence of mild inflammation of the intestinal mucosa during the challenge.

This potential diagnostic contribution of fecal calprotectin was not supported by subsequent studies. In fact, in a recent study by Roca et al. [82], fecal calprotectin levels in infants with NICMPA were higher than in healthy infants, even though the differences were not statistically significant. Moreover, after 1 month of an elimination diet, no statistically significant differences in fecal calprotectin with basal levels were observed [82].

Similarly, in a previous experience by Diaz et al. [83], fecal calprotectin concentrations in allergic infants with diagnosis of NICMPA, who were on an exclusion diet for six months, did not show statistical differences with those found in control infants.

To date, calprotectin levels were not a good test for predicting clinical response to milk withdrawal [84]. The authors studied 82 infants between 1 and 12 months of age, including 40 patients with NICMPA. They observed a statistically significant relationship (*p* < 0.0001) between high fecal calprotectin levels and infants suffering CMA at the time of diagnosis, at 1 month and 3 months (*p* < 0.001) from the elimination of CMP. However, fecal calprotectin levels had no power to predict a clinical response one month after CMP elimination [84].

#### 3.2.2. Food Protein-Induced Allergic Proctocolitis (FPIAP)

Food protein-induced allergic proctocolitis (FPIAP), formerly known as allergic or eosinophilic proctocolitis or “protein intolerance”, is a common problem in young infants [85,86,87]. Typical presenting symptoms of FPIAP are rectal bleeding, significant irritability, and diarrhea in an otherwise healthy infant [88]. It usually begins in the first weeks of life and, in most cases, resolves by late infancy [89]. FPIAP is characterized by inflammation of the distal colon in response to one or more food proteins, with no evidence of IgE involvement [90].

##### Granule Derived Alarmins: EDN

Studies on humans

Recently, Rycyk et al. [91] prospectively explored the potential role of three selected non-invasive, fecal biomarker (EDN, TNF-α, and calprotectin) measurements to improve the diagnosis of FPIAP in children. Fecal EDN concentration was significantly higher in children with FPIAP than in those with gastrointestinal functional disorders. Furthermore, a combined measurement of fecal calprotectin and EDN showed better diagnostic performance than testing each biomarker solely [91].

Similarly, de Boer et al. [92] measured EDN concentrations from rectal swabs and rectal biopsy samples in a group of 11 children (45% boys, mean age 6.9 months). EDN concentrations were elevated; both sampling methods revealed EDN concentrations significantly higher in the FPIAP group than in the control group (rectal biopsy: 183.6 ± 114.6 vs. 76.6 ± 71.0 μg/mL, *p* = 0.010; and rectal swab: 66.2 ± 64.8 vs. 20.4 ± 22.2 μg/mL, *p* = 0.025). This noninvasive test may be useful to screen for FPIP in children to confirm FPIAP diagnosis. [92].

##### Cytoplasmic Alarmins: S100, Calprotectin

Studies on humans

The detection of fecal calprotectin can support the diagnosis of FPIAP. In a prospective study [91], 27 infants with symptoms of hematochezia were enrolled, and FPIAP was confirmed by an open oral food challenge. Median calprotectin fecal levels were significantly higher in the study group than in the control group of infants with functional gastrointestinal disorders (*p* < 0.05), improving diagnostic performance if combined with fecal calprotectin [91].

#### 3.2.3. Food Protein-Induced Enterocolitis Syndrome (FPIES)

Food protein-induced enterocolitis syndrome (FPIES) is a type of non-IgE-mediated gastrointestinal FA, characterized by repetitive vomiting without classic IgE-mediated allergic skin or respiratory symptoms 1–4 h after causative food ingestion [93]. The disease is classified as acute or chronic, typical or atypical, and liquid or solid according to the course of symptoms, presence of IgE, and causative food, respectively [93].

##### Granule Derived Alarmins: EDN

Studies on humans

In 2014, Wada et al. [94] studied eight Japanese patients suffering from FPIES after a period of trigger-foods elimination diet. Fecal samples were collected before and after the food challenge test and compared with a control group of 12 healthy infants. A significant increase in fecal EDN was demonstrated in all patients (mean, 33,244 ng/mL) with median time to maximum concentration of 23.5 h [94].

Two years later, the same researchers explored the expression of activation marker CD69 on circulating eosinophils from five patients with FPIES [95]. Consistent with their previous report [94], a significant increase in fecal EDN on the day after ingestion of the causative food was observed in all patients (mean 26,670 ng/mL), with a median time to maximum concentration of 30 h [95].

##### Cytoplasmic Alarmins: S100, Calprotectin

Studies on humans

In the previously mentioned study by Wada et al., the ingestion of causative food induced a significant increase in fecal calprotectin levels, although it was much less than fecal EDN [94].

### 3.3. Mixed FA

#### 3.3.1. Eosinophilic Esophagitis

Eosinophilic esophagitis (EoE) is an allergic inflammatory disorder characterized by accumulation of eosinophils in the esophagus [96,97,98,99]. EoE often coexists with atopic dermatitis, a chronic inflammatory skin disease. The impaired skin barrier in patients with atopic dermatitis has been suggested as an entry point for allergic sensitization that triggers the development of EoE [100].

##### Nuclear Alarmins: TSLP and IL-33

Studies on animal models

In 2016, Venturelli et al. [101] observed that epicutaneous allergic sensitization promoted EoE that was mediated through the IL-33/ST2–basophil axis. In particular, the authors epicutaneously sensitized mice with OVA and then performed intranasal OVA challenge. Epicutaneous sensitization and intranasal challenge of wild-type mice resulted in accumulation of eosinophils and upregulation of TH2 cytokines and St2 in the esophagus. Disruption of the IL-33–ST2 axis or depletion of basophils reduced these features. The expression of ST2 on basophils was required to accumulate in the esophagus and transfer experimental EoE [101]. In a previous study, authors concluded that the development of FA was promoted by the accumulation of TSLP-elicited basophils derived from epicutaneous sensitization in mice epicutaneously sensitized with OVA or peanut on an AD-like skin lesion, followed by intragastric antigen challenge [38].

Similarly, exogenous IL-33 application provides a mechanistic rationale for EoE development in mice, promoting transmural eosinophilia, mucosal hyperproliferation, upregulation of eosinophilic genes and chemokines, and the inhibition of the regulatory T cell with loss of antigenic tolerance [102].

Studies on humans

The evidence that EoE is associated with impaired barrier function emerged in 2015 [103]. Simon et al. investigated the presence and distribution of eosinophil extracellular traps (EETs) able to kill bacteria in esophageal tissues from EoE patients. EET formation occurred frequently and was detected in all EoE samples correlating with the numbers of infiltrating eosinophils. The researchers also observed that the expression of alarmins TSLP, IL-25, and IL-33 were elevated in EoE compared to normal esophageal tissues; moreover, a significant correlation between EET formation and TSLP expression was observed (*p* = 0.02) [103].

A correlation between EoE genotype–phenotype through the evaluation of TSLP risk alleles was found by Fahey et al. [104] in a retrospective study on 309 children. The authors analyzed the phenotypic characteristics in EoE children with the TSLP EoE risk allele. The number and type of EoE food allergen triggers were compared with genotype using chi-square analysis. An increase in the number of patients with three or more EoE food allergens was noted among those who were either homozygous or heterozygous for the risk allele compared to those without the risk allele (*p* < 0.0001). Moreover, patients homozygous for the risk allele had greater TSLP secretion than those isolated from heterozygous patients [104].

Previously, a study on genome-wide association by Rothenberg et al. [105] identified the genetic susceptibility locus of EoE on 5q22, a region that harbors the TSLP gene, and SNP rs3806932 was significantly associated with EoE.

In the same year, Sherrill et al. [106] identified an SNP rs10062929 residing in the TSLP gene reaching statistically significance only when EE cases were compared to allergic controls. A non-synonymous polymorphism in the TSLP receptor on Xp22.3 and Yp11.3 was significantly associated with disease only in male EE patients [106].

In addition to the role of TSLP, the IL-33/STR2 axis appears to be fundamental in the pathogenesis of EoE in humans [107]. Uchida et al. reported frequent expression of the IL-33 receptor ST2 on esophageal-infiltrating eosinophils compared to blood eosinophils, other granulocytes, and Th2 cells. Eosinophils that lack antigen-specific T cell receptors maintained their ability to respond to IL33. This may explain the continued inflammation observed in patients with EoE after removal of dietary antigen [107].

Nonetheless, the experience of Ishihara et al. [108] focused on the possible value of eosinophil-related proteins as serum biomarkers of EoE in 29 patients and 80 controls. Their research did not show adequate sensitivity to allow the use of the proteins investigated as biomarkers for the diagnosis or monitoring of eosinophilic gastrointestinal diseases.

##### Granule Derived Alarmins: Defensins (α, β)

Studies on humans

Defensins are antimicrobial peptides expressed on mucosal surfaces that contribute to maintaining intestinal homeostasis by providing innate defense mechanisms for the epithelia. Defensin expression is altered in several diseases that affect mucosal surfaces, such as AD, allergic rhinitis, and inflammatory bowel disease. Similarly, EoE is a chronic disease in which the squamous epithelial surface is affected by a similar Th2 microenvironment and eosinophil predominant inflammation. Research on the possible role of defensins in EoO is ongoing.

In 2015, Simon et al. [103] analyzed tissue samples from 18 patients with active EoE and observed that epithelial antimicrobial peptides such as human beta-defensin-2 and human beta-defensin-3 were elevated in EoE compared to normal esophageal tissues.

Differently, a previous study by Schroeder et al. [109] revealed that diminished expression of hBD1 and hBD3 could make the esophageal epithelium more susceptible to the development and/or perpetuation of EoE; the authors observed that specimens from EoE pediatric patients revealed a significant decrease in mRNA and protein expression for hBD1 and hBD3 compared to esophageal biopsy specimens from gastroesophageal reflux disease and control children [109].

**Table 1:** Human studies on nuclear alarmins in food allergy.

Alarmins	FA Type	Food	Study Design	Age (Year/Months)	F/M	N° of Patients	Country	Year [Ref.]
**TSLP**	IgE	Shrimp	Case-Control	42ys	19/18	37	Poland	2021 [48]
	IgE	Peanut	Multi-omic	10ys	17/39	56	USA	2021 [50]
	Mixed (EE)		Cross-sectional	Unknown		18	Switzerland	2015 [103]
	Mixed (EE)		Retrospective	Unknown	56/253	309	USA	2018 [104]
	Mixed (EE)		Cohort	10ys	96/255	351	USA	2010 [105]
	Mixed (EE)		Cohort	9ys	54/118	172	USA	2010 [106]
**IL-33**	IgE	Shrimp	Case-Control	42ys	19/18	37	Poland	2021 [48]
	IgE	Peanut	Case-Control	10ys	17/39	56	USA	2021 [50]
	Mixed (EE)		Cross-sectional	Unknown		18	Switzerland	2015 [103]
	Mixed (EE)		Cross-sectional	39ys	35/40	75	USA	2022 [107]
	Mixed (EE)		Cross-sectional	48ys	10/19	29	Japan	2017 [108]
**IL-25**	IgE	Shrimp	Case-Control	42ys	19/18	37	Poland	2021 [48]
	IgE	Peanut	Cross-sectional	8ys	8/22	30	Netherlands	2013 [51]
	Mixed (EE)		Cross-sectional	Unknown		18	Switzerland	2015 [103]
**IL-1α**	Not evaluated							
**HMGB1**	Not evaluated							
**HMGN1**	Not evaluated							

FA, food allergy; TSLP, thymic stromal lymphopoietin; EE, eosinophilic esophagitis; HMGB1, high-mobility group box 1 protein; HMGN1, high-mobility group nucleosome-binding domain 1 protein.

**Table 2:** Human studies on granule-derived alarmins in food allergy.

Alarmins	FA Type	Food	Study Design	Age (Year/Months)	F/M	N° of Patients	Country	Year [Ref.]
**Cathelicidin (LL37/CRAMP)**	Not evaluated							
**Defensins (α, β)**	Non-IgE	Cow’s milk	Cross-sectional	9ms	Unknown	57	Finland	2014 [79]
	Mixed (EE)		Cross-sectional	41ys	2/16	18	Switzerland	2015 [103]
	Mixed (EE)		Cross-sectional	10ys	11/11	22	USA	2013 [109]
**EDN**	IgE	Cow’s milk, egg, cod	Cohort	Unknown	6/2	8	Sweden	2016 [55]
	IgE	Cow’s milk	Longitudinal	17ys	14/10	24	Finland	2018 [56]
	Non-IgE (NICMPA)	Cow’s milk	Cross-sectional	1m	14/16	30	Spain	2021 [82]
	Non-IgE (NICMPA)	Cow’s milk	Case report	Newborn	1/0	1	Japan	2007 [80]
	Non-IgE (FPIAP)	Cow’s milk	Cross-sectional	4ms	9/18	27	Poland	2020 [91]
	Non-IgE (FPIAP)		Prospective, open-label pilot	7ms	6/5	11	USA	2020 [92]
	Non-IgE (FPIES)	Fish, cow’s milk, egg, wheat, rice	Cross-sectional	3ms	5/3	8	Japan	2014 [94]
	Non-IgE (FPIES)	Fish, egg, wheat, rice	Cross-sectional	3ms	3/2	5	Japan	2016 [95]
**Granulysin**	Not evaluated							

FA, food allergy; CRAMP, cathelicidin-related antimicrobial peptide; EE, eosinophilic esophagitis; EDN, eosinophil-derived neurotoxin.

**Table 3:** Human studies on cytoplasmic alarmins in food allergy.

Alarmins	FA Type	Food	Study Design	Age (Year/Months)	F/M	N° of Patients	Country	Year [Ref.]
***Cytoplasmic***								
**S100 proteins (calprotectin)**	IgE	Cow’s milk	Case–Control	0–9ms	49/41	90	China	2021 [65]
	IgE	Cow’s milk	Longitudinal	33ys	127/33	160	Italy	2011 [66]
	IgE	Cow’s milk, Egg, Cod	Cohort	Unknown	6/2	8	Sweden	2016 [55]
	Non-IgE (NICMPA)	Cow’s milk	Cross-sectional	9ms	3/5	8	Turkey	2014 [81]
	Non-IgE (NICMPA)	Cow’s milk	Cross-sectional	8ms	Unknown	18	Finland	2014 [79]
	Non-IgE (NICMPA)	Cow’s milk	Cross-sectional	1m	14/16	30	Spain	2021 [82]
	Non-IgE (NICMPA)	Cow’s milk	Prospective cohort	12–24ms	8/9	17	Spain, Italy	2018 [83]
	Non-IgE (NICMPA)	Cow’s milk	Cross-sectional	4ms	15/25	40	Spain	2016 [84]
	Non-IgE (FPIAP)	Cow’s milk	Cross-sectional	4ms	9/18	27	Poland	2020 [91]
	Non-IgE (FPIES)	Fish, cow’s milk, egg, wheat, rice	Cross-sectional	3ms	5/3	8	Japan	2014 [94]
**HSP**	IgE	Milk	Comparative	53ys	13/13	26	USA	2002 [70]
	IgE	Peanut, soy, tree nuts, crab, clam, lobster, egg.	Cross-sectional, proteomic analysis	8ys	5/6	11	USA	2011 [71]
**ATP**	Not evaluated							
**Uric acid**	IgE	Cow’s milk	Survey	Unknown	64/87	151	Republic of Korea, USA *	2016 [77]

CRAMP, cathelin-related antimicrobial peptide; EDN, eosinophil-derived neurotoxin; HMGB1, high-mobility group box 1 protein; HMGN1, high-mobility group nucleosome-binding domain 1 protein; HSP, heat-shock proteins; ATP, adenosine 5′-triphosphate; *, adult population from USA.

## 4. Discussion

In the literature, the role of alarmins in the genesis of IgE-mediated food allergies has been demonstrated by various clinical studies on mouse models and, subsequently, humans. Mouse models deficient in TSLPR have shown a lower clinical response to allergen exposure than TSLPR+/+ mice, and a reduced expression of food-specific IgE [31]. Furthermore, mice sensitized to OVA or peanut showed an increase in TSLP-activated basophils, with a consequent increase in specific serum IgE and mast cells after intragastric challenge [32]. TSLP appears to be essential during the sensitization phase in inducing the Th2 response, similarly to IL-25 [38,39]. IL-25 receptor-deficient mice tend not to develop diarrhea and anaphylaxis after challenge tests [39]. Instead, IL33 seems to have a role during early skin inflammation and in driving anaphylaxis, favoring mast cell degranulation [34,38].

Calprotectin appears to promote inflammation in OVA-sensitized rat models [44,45].

Studies on humans confirmed the importance of alarmins in IgE-mediated allergies: serum concentrations of TSLP and IL-25 are found to be increased in patients with food allergy [40], and IL-25 levels appear to correlate positively with atopic severe phenotypes [43]. TSLP and IL-33 could also play a role in the development of tolerance in humans [41], and their expression in the oral mucosa would be influenced by the microbiome [42]. Fecal calprotectin levels appear to be significantly elevated in patients with food allergies, which decrease after an exclusion diet [46].

In the study of non-IgE-mediated FA, many authors have focused on the possible diagnostic and prognostic value of calprotectin. However, evaluating fecal calprotectin levels in patients with NICMPA has given conflicting results [50,51,52,53,54]. Furthermore, the evaluation of fecal beta-defensin 2 levels did not provide statistically significant results [49]. Instead, fecal calprotectin levels can support diagnosing FPIAP and FPIES, especially when associated with determining eosinophil-derived neurotoxin levels [61,62]. However, studies in this regard are limited, and they are based on restricted patient cohorts.

Alarmins also play a role in mixed-type adverse food reactions, such as EoE. In mouse models of EoE, the IL-33/ST2-basophil axis plays a crucial role in promoting inflammation; blockade of this axis reduces eosinophilic infiltration and TH2 cytokine levels [98]. These findings have also been confirmed in studies on humans [104], in which increased IL-33, TSLP, and IL-25 were found [101]. Furthermore, mutations in the gene encoding TSLP drive disease susceptibility and are associated with a major risk of food allergy development [101,102,103]. Finally, the role of defensins in EoE has still to be determined due to there being few studies and conflicting results.

Our literature review highlighted the potential role of alarmins in the pathogenesis of FA. Cytoplasmic and granule-derived alarmins could become biomarkers of disease and the response to an exclusion diet. The role of TSLP, IL-33, and IL-25 in the pathogenesis of the different FAs is fundamental; therefore, they could represent a therapeutic target. Khodoun et al. demonstrated in mouse models that the use of monoclonal antibodies (mAbs) directed against TSLP, IL-33R, or IL-25 blocks the development of FA. However, a single mAb cannot suppress an already-developed FA. Therefore, an established FA may need a cocktail of the three mAbs [43]. The available data are promising, but studies in this regard are still few and, in some cases, have conflicting results. Many of these have been carried out on animal models and have never been applied to humans. FAs are pathologies with various pathogenetic mechanisms that can significantly alter the patient’s quality of life, even being lethal on some occasions, and for which effective therapies do not yet exist. More insights and tests on human models could lead to the development of revolutionary therapeutic strategies with the aim of managing food allergies. Finally, studies on larger cohorts could favor the identification of biomarkers useful for diagnostic and therapeutic purposes.

## Data Availability

Not applicable.
